# Network-based drug repurposing for schizophrenia

**DOI:** 10.1038/s41386-024-01805-6

**Published:** 2024-02-06

**Authors:** Trang T. T. Truong, Zoe S. J. Liu, Bruna Panizzutti, Jee Hyun Kim, Olivia M. Dean, Michael Berk, Ken Walder

**Affiliations:** 1 Deakin University, IMPACT, The Institute for Mental and Physical Health and Clinical Translation, School of Medicine, Geelong, Australia; 2 Florey Institute of Neuroscience and Mental Health, Parkville, Australia; 3 Orygen, The National Centre of Excellence in Youth Mental Health, Centre for Youth Mental Health, The Florey Institute for Neuroscience and Mental Health and the Department of Psychiatry, University of Melbourne, Parkville, 3010 Australia

**Keywords:** Drug development, Transcriptional regulatory elements

## Abstract

Despite recent progress, the challenges in drug discovery for schizophrenia persist. However, computational drug repurposing has gained popularity as it leverages the wealth of expanding biomedical databases. Network analyses provide a comprehensive understanding of transcription factor (TF) regulatory effects through gene regulatory networks, which capture the interactions between TFs and target genes by integrating various lines of evidence. Using the PANDA algorithm, we examined the topological variances in TF-gene regulatory networks between individuals with schizophrenia and healthy controls. This algorithm incorporates binding motifs, protein interactions, and gene co-expression data. To identify these differences, we subtracted the edge weights of the healthy control network from those of the schizophrenia network. The resulting differential network was then analysed using the CLUEreg tool in the GRAND database. This tool employs differential network signatures to identify drugs that potentially target the gene signature associated with the disease. Our analysis utilised a large RNA-seq dataset comprising 532 post-mortem brain samples from the CommonMind project. We constructed co-expression gene regulatory networks for both schizophrenia cases and healthy control subjects, incorporating 15,831 genes and 413 overlapping TFs. Through drug repurposing, we identified 18 promising candidates for repurposing as potential treatments for schizophrenia. The analysis of TF-gene regulatory networks revealed that the TFs in schizophrenia predominantly regulate pathways associated with energy metabolism, immune response, cell adhesion, and thyroid hormone signalling. These pathways represent significant targets for therapeutic intervention. The identified drug repurposing candidates likely act through TF-targeted pathways. These promising candidates, particularly those with preclinical evidence such as rimonabant and kaempferol, warrant further investigation into their potential mechanisms of action and efficacy in alleviating the symptoms of schizophrenia.

## Introduction

Drug discovery for schizophrenia continues to be a formidable challenge despite recent pharmacological advances. Most effective antipsychotics currently available were discovered via clinical observations and serendipity more than 60 years ago [1]. Without credible biomarkers as well as animal models adequately representing the disease, the complexity of schizophrenia makes drug development, which is already a laborious process, all the more challenging [2, 3].

As an alternative to conventional drug discovery, drug repurposing has recently gained popularity. Considering known safety profiles and bioavailability, as well as established manufacturing processes, drug repurposing can bypass several steps compared to conventional drug discovery, thereby reducing the cost and risk of the development process [4, 5]. A variety of computational drug repurposing approaches have facilitated novel treatment research strategies by taking advantage of expanding biomedical databases.

Recently, network analysis – the use of multiple layers of knowledge to identify latent connections between components has emerged as a powerful tool for drug discovery. A recent example is integrating the human interactome with viral and drug targets to find repurposing medications for COVID-19 [6, 7]. Fitting well with the “one drug multiple targets” or poly-pharmacology paradigm shift in drug discovery for complex psychiatric disorders, a network medicine framework allows a simultaneous and comprehensive view of various biological components and their relationships [8–10].

Transcriptomics has been an essential feature of the genomic landscape and offers a comprehensive reflection of molecular status related to pathophysiology and medication effects [11, 12]. In this context, transcription factors (TFs) – as regulators of gene expression, play a major role in driving pathological conditions. Previous studies have highlighted the importance of exploring the main drivers of transcriptional profiles over the simple evaluation of all differentially expressed genes to explore the mechanism of phenotypic transitions [13, 14]. While the impact of gene expression regulators is amplified by the cascade of downstream targets, such regulatory influence is affected by not only the regulators’ expression level but also the availability of co-factors and targets as well as post-translational modifications. Hence, TFs’ activities may not coherently correlate directly to their expression levels and should be considered with other interacting elements, particularly their targets [15]. Recent systems-level analyses allow the comprehensive assessment of TF regulatory effects via gene regulatory networks, reflecting TF and target genes interactions by incorporating multiple lines of evidence complementing gene expression such as motif binding and physical protein interactions [16–18].

In this study, we first identified the topological differences of the TF-gene regulatory networks of schizophrenia cases versus healthy controls using PANDA (Passing Attributes between Networks for Data Assimilation). PANDA uses information from different data types (i.e., motifs, protein interactions, gene co-expression) to iteratively refine predictions of context-specific regulatory relationships by searching for agreement among available evidence [16]. By focusing on the differential interactions (edges of the network), PANDA highlights meaningful patterns in regulatory changes for genes that are not differentially expressed [19]. These perturbations can then be utilised as network-based signatures for finding potential drug repurposing candidates for the treatment of schizophrenia.

The notion of signatures for drug repurposing was based on Connectivity Map (CMap) [20] and the Library of Integrated Network-based Cellular Signatures (LINCS) [21], where transcriptional expression patterns are considered as the unique ‘signature’ of disease states as well as drug effects [22, 23]. By matching signatures based on their dissimilarity or similarity, potential drug-disease connections (signature reversion strategy) or drug-drug associations (guilt-by-association strategy) respectively can be explored and interrogated for drug repurposing [23]. Typical signature-based approaches on differential expression profiles have several limitations: differential expression profiles are susceptible to poor reproducibility [18, 21] and simplistic signature matching ignores the interactions between genes and their functional redundancy [24]. Network-based approaches which consider modular units as key regulators instead of a single set of individual genes could offer a more biologically relevant approach to mitigate these limitations. Integration of more data sources and network models can not only improve reproducibility and robustness but also yield more biologically relevant insights into molecular mechanism(s) at a systems level [18, 24, 25]. Therefore, our application of gene regulatory networks could shed light on biologically important processes associated with numerous phenotypes, which may be missed when looking at gene expression alone. To our best knowledge, this is the first-time gene regulatory networks were used for drug repurposing for schizophrenia.

## Methods

### RNA sequencing data

Dorsolateral prefrontal cortex (DLPFC) RNA sequencing data were accessed from the CommonMind Consortium [26]. After quality control, a total of 532 post-mortem samples belonging to the MSSM – Pitt – Penn Brain Bank were collected from 279 healthy control subjects and 253 people with schizophrenia. Genes being expressed at more than 0.5 count per million (CPM) in at least 30% of samples were kept for downstream analyses. While within- and between-sample normalisations are commonly used for gene expression analyses such as differential expression, a comprehensive benchmark study of normalisation techniques for co-expression network construction by Johnson et al. found that any normalisation mainly results to worse performance than not using it [27]. Therefore, in this study, no normalisation was applied to the read counts given the lack of evidence justifying its use in network construction.

The R package variancePartition was used to produce expression residuals as input for the co-expression network [28]. We accounted for covariates with the most variance explained and/or the greatest spreads in the linear mixed model as shown in Supplementary Fig. 1 (i.e., diagnosis, sex, RNA integrity number, cell type composition, institution, age of death, intronic rate, intragenic rate, intergenic rate, ribosomal RNA rate). These covariates were regressed out (i.e., we excluded the effects by such variables), followed by the adding back of main variable of diagnosis and the intercept. The expression residuals were pre-processed (removal of genes with no counts, taking the average of duplicated genes) before being calculated for co-expression in PANDA using Pearson correlations.

### Gene co-expression regulatory networks

The R package PANDA was used to build the bipartite gene regulatory network that linked TFs to their target genes via a guilt-by-association approach with two main scenarios: (1) if TF A was known to regulate gene B, then TF A may regulate gene C which is co-expressed with gene B; (2) if TF X regulates gene Y then a TF Z interacting with TF X may also co-regulate gene Y [16]. PANDA integrates three sources of information to infer the TF-gene regulatory network: TF physical protein-protein interactions (TF - TF links), gene co-expression (gene - gene links) and TF motif binding sites (TF - gene links) [16].

TF protein-protein interactions (PPI) were obtained from the STRING database [29] with confidence scores reflecting how likely an interaction was considered to be true from combined sources of evidence. A threshold of 0.7 (high confidence) was applied to the combined score to convert the score to binary (0 implies no interaction and 1 implies high likelihood of interaction). Binding motifs were acquired from previous studies [30, 31], where TF binding domain sequences (i.e., motifs) were scanned for their presence in the promoter regions of genes where transcription initiates.

Expression residuals, TF PPI and binding motifs were inputted in PANDA with the following non-default parameters to make sure only mutual connections shared by PPI, co-expression and TF motifs were considered in the networks: mode = “legacy”, remove.missing.motif = True, remove.missing.ppi = True, remove.missing.genes = True. Two separate regulatory networks were built for schizophrenia cases and healthy control subjects. Edge weight of each network implied the strength of connection of TFs and genes, reflected via Pearson’s correlation coefficient between the TF and the target gene.

### Differential schizophrenia network

To find the differences in regulation in schizophrenia patients as compared to healthy control subjects, the two corresponding regulatory networks were first aligned and filtered to keep intersections of genes and TFs only. Then the differential network was estimated by subtracting the edge weights of the healthy control network from those of schizophrenia network. All networks were imported and visualised in Cytoscape [32].

Gene regulatory network analysis is based on the hypothesis that alterations in the way TFs regulate genes lead to “targeting” patterns that explain phenotypic perturbations or reactions to specific stimuli. When conducting comparative gene regulatory network analysis, TFs that regulate gene sets with different patterns (e.g., changing targets, disturbance in the order of targeting intensity) in the compared phenotypes are typically identified as “differential targeting” TFs [33]. Enrichment analysis of differential targeting was performed based on Subramanian et al. rank-based gene set enrichment analysis (GSEA) [34] via the R package ClusterProfiler [35], with gene lists ranked based on differential targeting score and pathway reference from the Kyoto Encyclopedia of Genes and Genomes (KEGG) database [36].

Wilcoxon signed-rank test was applied on the non-normally distributed targeting scores (Shapiro–Wilk normality test) to identify TFs with significant differential targeting between schizophrenia and healthy control subjects. To account for multiple testing correction, Benjamini-Hochberg adjusted *q*-values were generated.

### Finding drug repurposing candidates

The 100 top positively differential TFs and 100 top negatively differential TFs based on the differential targeting score were submitted to the CLUEreg tool of the GRAND database [33] which utilises differential network signatures to find drugs that potentially target the disease’s gene signature. The drug typically is expected to revert the abnormal alterations to normality, knowing as “signature reversion” approach in signature-based drug repurposing [23]. Herein, ideal drug matches are the ones that negatively regulate the top 100 positively regulated TFs in samples with schizophrenia, and positively regulate the top 100 negatively regulated TFs in schizophrenia. The similarity of a pair of network-based signatures was evaluated by cosine similarity score and statistical significance was calculated to compare such score with those of other pairs. The more negative cosine value suggests the drug’s signature is more dissimilar to the disease, suggesting higher likeliness of reversing the queried disease.

## Results

Using a large RNA-seq dataset of 532 post-mortem brain samples [26], we built co-expression gene regulatory networks for schizophrenia cases and healthy control subjects. Patient clinical features of samples used in the RNA-sequencing are in Supplementary Table 1. Each network was pruned to retain 15,831 genes and 413 TFs overlapping in both schizophrenia and healthy control networks. In corresponding networks for each phenotype (Supplementary Fig. 2 for schizophrenia cases and Supplementary Fig. 3 for healthy controls – due to limited space only top 200 edges for each network were illustrated), an edge connecting a TF to its target gene reflects the likelihood of the regulatory relationship. The edge weight was represented by the z-score of the confidence interval calculated by PANDA [16].

**Fig. 1:**
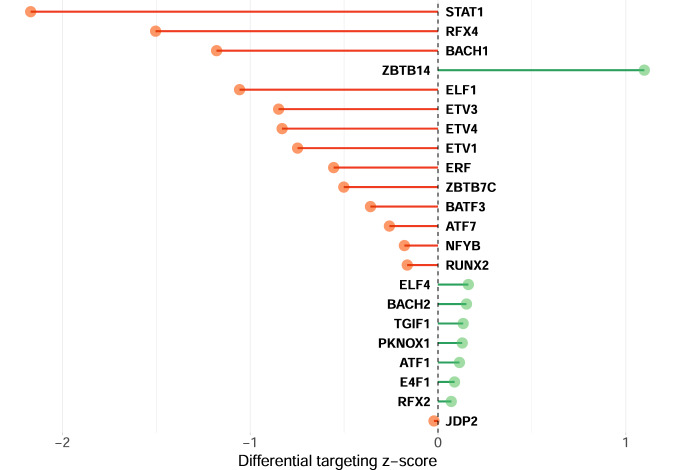
Transcription factors with significant differential targeting between schizophrenia and healthy control subjects. Statistical significance was set as Benjamini-Hochberg adjusted *q*-values < 0.05. Green lines imply increased targeting or increased regulatory effects on respective genes (positive z-score) in schizophrenia. Red lines represent decreased targeting or decreased regulatory effects on corresponding genes (negative z-score) in schizophrenia.

**Fig. 2:**
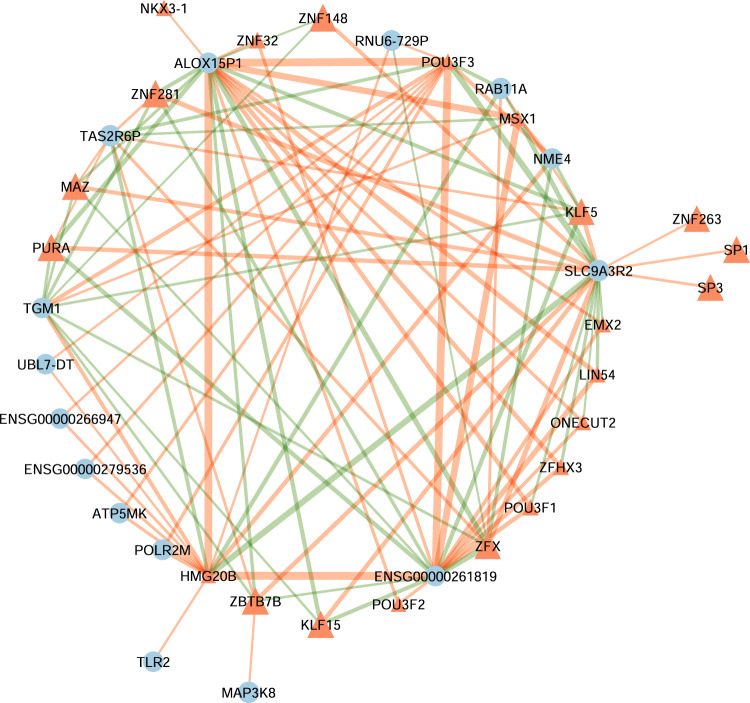
Top 100 differential connections of the differential gene regulatory network of schizophrenia versus healthy control. Orange triangles are transcription factors and blue circles are their targeted genes. Node sizes are proportional to their targeting score. Green edges imply increased targeting in schizophrenia, red edges represent decreased targeting in schizophrenia. Edge weights/thickness are proportional to the absolute differential targeting of corresponding connections.

To find topological differences between the gene regulatory networks of schizophrenia versus healthy controls, we subtracted the edge weights of the healthy control network from those of the schizophrenia network. TFs with significant differential targeting scores between schizophrenia and healthy control subjects were represented in Fig. 1. Bar plot of variance partitioned on the variables accounted in variancePartition’s linear mixed model for these TFs is presented in Supplementary Fig. 4. The network with the top 100 differential regulatory edges sorted by largest absolute value is shown in Fig. 2. Sum of edge weights was used as a summary measure for each node (i.e., gene or TF). The term “gene targeting” implies the weighted in-degree of each gene (i.e., the sum of the incoming edge weights from all TFs in the network to that gene), and “TF targeting” implies the weighted out-degree for each TF (the sum of outgoing edges from that TF to all its target genes).

**Fig. 3:**
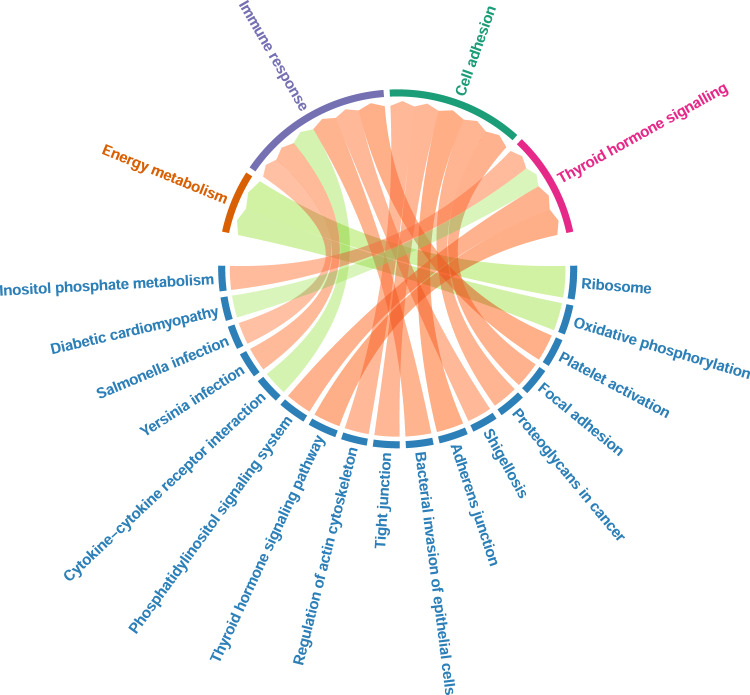
Grouping of significantly enriched KEGG pathways. Significantly enriched KEGG pathways (*q*-value < 0.05) are blue labels at the bottom half of the chord diagram, with links grouping them to four main biological functions at the top half. KEGG pathways are ordered clock-wisely as per significance of *p*-values. Green links imply increased targeting in schizophrenia, red links represent decreased targeting in schizophrenia.

The gene targeting difference between the schizophrenia and healthy control networks was then used as ranking metric for enrichment analysis of differential targeting. Significant pathways are presented in Fig. 3. The full enrichment results can be found in Supplementary Table 2. Positive normalised enrichment score (NES) implies more TF targeting and negative NES implies less TF targeting on the corresponding pathway in schizophrenia. Ribosome and oxidative phosphorylation were most positively targeted pathways by TFs, while platelet activation and focal adhesion were most negatively targeted pathways.

From the differential network, the 100 top positively differential TFs and 100 top negatively differential TFs were then used as network-based signatures to query for potential drug repurposing candidates for schizophrenia. From the top 100 drug repurposing candidates highlighted by the GRAND database, we focused on drugs having Unique Ingredient Identifier (UNII) generated by US Food and Drug Administration [37], that have known activities in the central nervous system, have been approved or are undergoing clinical trials (Table 1). The full results with relevant literature review evidence can be found in Supplementary Table 3.

**Table 1:** Shortlisted drug repurposing candidates for the treatment of schizophrenia.

Drug	Cosine	*Q*-value	Pharmacological targets	Therapeutic indication
Alendronic-acid	−0.3871	<0.001	Farnesyl diphosphate synthase - InhibitorGeranylgeranyl pyrophosphate synthetase - Inhibitor Acetylcholinesterase - Inhibitor	Glucocorticoid-induced osteoporosis - Approved Osteoporosis - ApprovedPaget’s disease - Approved
Khellin	−0.3446	<0.001	Cytochrome P450 1A1 - Inhibitor Aryl hydrocarbon receptor - Activator Ca2+ influx - Inhibitor	Angina pectoris - ApprovedAsthma - Approved Vitiligo - Phase II
Rimonabant	−0.3203	<0.001	Cannabinoid CB1 receptor - Inverse Agonist Cannabinoid CB2 receptor - Inverse Agonist	Obesity - ApprovedArteriosclerosis - Phase IIIFatty liver disease - Phase III
Kaempferol	−0.3165	<0.001	Ribosomal protein S6 kinase alpha 5 - Inhibitor DNA topoisomerase II - Inhibitor Monoamine oxidase A - Inhibitor Ribosomal protein S6 kinase alpha 3 - Inhibitor	Osteoarthritis - Phase II Cancer - Preclinical Depression - Preclinical
Alizapride	−0.3008	<0.001	Dopamine D2 receptor - Antagonist	Nausea and vomiting - Approved
Glutamine	−0.2953	<0.001	Protein-glutamine gamma-glutamyltransferase - Substrate CTP synthase 1 - Antagonist Apoptotic process - Inhibitor Glutaminase kidney isoform, mitochondrial - Substrate	Short bowel syndrome - Approved
Carbachol	−0.2939	<0.001	Muscarinic acetylcholine receptor - Agonist Acetylcholinesterase - Substrate	Elevated intraocular pressure - Approved
Vidarabine	−0.2821	0.0271	Adenosine receptor - Agonist Thymidine kinase - Substrate Human herpesvirus 1 DNA polymerase - Inhibitor	Paroxysmal supraventricular tachycardia - ApprovedKeratoconjunctivitis - Approved Epithelial keratitis - ApprovedHerpes simplex infection - Phase III
Ellagic-acid	−0.2738	<0.001	Tyrosine-protein kinase TIE-2 - Inhibitor Aldose reductase - Inhibitor Casein kinase II alpha - Inhibitor	Follicular lymphoma - Phase II HPV infection - Pilot randomised controlled trial
Benfotiamine	−0.2321	0.0317	Glycogen synthase kinase-3 - Inhibitor	Type 1 diabetes mellitus - Phase IIAlzheimer’s disease - Phase II

Cosine: Similarity of drug versus disease, more negative is better (more dissimilar). Q-value: Benjamini-Hochberg corrected p-value. Phase refers to the current clinical trial stage.

As a validation for drug repurposing results, we applied the similar analyses using two independent post-mortem datasets as replications of the main dataset: HBCC Brain Bank from CommonMind Consortium, BrainGVEX study from PsychENCODE [26, 109]. A Supplementary Methods and Results. We found 49 out of 100 repurposing candidates with statistical significance (*q*-value < 0.05) from the current dataset (MSSM – Pitt – Penn Brain Bank from CommonMind Consortium) were replicated in both datasets. Only 6 drugs were not replicated in any analysed datasets. All shortlisted drugs in Table 1 were replicated in at least one dataset, with 6 candidates (alendronic-acid, rimonabant, alizapride, glutamine, carbachol, ellagic-acid) being replicated in both datasets.

## Discussion

The current study deployed network analyses to identify different targeting patterns of TFs in schizophrenia versus healthy controls. We then applied the acquired TF signatures of differential network targeting for drug repurposing. While TFs are generally expressed at lower levels than non-TF genes, their effects may be amplified by the cascade of regulatory mechanisms they induce [38]. The use of differential targeting enabled the comparison of the flow of regulation rather than the state of single genes as in differential expression, where TFs could be less prioritised than their potential targets with higher expression [39]. Herein, we found our most differentially targeting TFs (Fig. 1) were not the most differentially expressed genes highlighted by a previous study by Fromer et al. using a similar dataset [40]. However, these TFs have been associated with schizophrenia in other studies as shown in Supplementary Table 5. Moreover, no significantly enriched pathways by differentially expressed genes were observed in Fromer et al., while we identified some pathways enriched by the differential targeting. Interestingly, our enrichment analysis of differential targeting highlighted several main biological functions (Fig. 3), i.e., energy metabolism, immune response, cell adhesion and thyroid hormone signalling, which are highly relevant to schizophrenia.

Impaired energy metabolism has been reported in schizophrenia, mainly owing to mitochondrial dysfunction [41, 42]. Mitochondria engage in oxidative phosphorylation, which is the main energy-producing pathway [43]. There have been abnormalities reported in schizophrenia in the gene expression and activity of oxidative phosphorylation complexes, mostly of complex I, affecting the production of high-energy phosphates [44]. Positive symptomology and active psychosis are associated with increased complex I activity, whereas residual psychosis is associated with decreased activity [45]. Antipsychotics have been shown to decrease oxidative phosphorylation and related respiratory responses in different neuronal cell models, potentially via complex I [46, 47]. For ribosomes, increased total protein levels and protein synthesis were reported in induced pluripotent stem cells derived from schizophrenia patients versus healthy controls [48]. We also found antipsychotic drugs reduced overall expression of ribosomal genes and protein synthesis in neuronal-like cells [49]. The validity of this finding is suggested by research showing that N-acetylcysteine which ameliorates redox dysfunction may have benefits in schizophrenia, especially negative symptoms [50].

The immune response has been associated with schizophrenia, given many risk genes of the disorder also play roles in inflammation and pathogen life cycles [51, 52]. Such links support the hypothesis of schizophrenia being a pathogenetic autoimmune disease: pathogen-induced knockdown may contribute to the immune activation in the patient’s brain and lymphocytes, as well as immune-related gene variants in schizophrenia [53, 54]. While there are contradictory results regarding the direction of cytokine level changes that could result from different disease stages and patient conditions, disturbances in cytokine levels and interactions may be significant contributors to schizophrenia pathophysiology [55]. Agents affecting inflammation such as minocycline and celecoxib have been explored in schizophrenia with variable results [56–58].

Cell adhesion is a major contributor to maintaining neuronal structure and regulates synaptic plasticity, as well as complex brain functions such as memory and learning [59]. In the developing nervous system, disrupted neuronal cell adhesion can cause neural circuits to malfunction, potentially leading to several neuropsychiatric diseases including schizophrenia [60]. Integrins, cadherins and claudins are among the main groups of cell adhesion molecules and are linked via the actin cytoskeleton. Cadherins are responsible for homotypic adhesion between cells (forming adherens junctions), integrins are responsible for adhesion between the cell and its extracellular matrix (contributing to focal adhesion), and claudins form the tight junction regulating paracellular barrier permeability [61, 62]. Proteoglycans provide a contact link between the cell membrane and the surrounding extracellular matrix [63]. Abnormalities of these elements have been reported in schizophrenia: reduced focal adhesion in patient-derived cells [64], negative correlation between expression of tight junction mRNAs and disease duration [65], and loss of adherens junctions in human iPSC-derived neural progenitors carrying a risk variant [66]. Altered levels of immune cell adhesion molecules in the plasma of schizophrenia patients also suggested the link of disrupted cell adhesion to abnormal immunomodulation in the disorder as discussed previously [67, 68].

Thyroid hormones have been known to play a vital role in neuronal and glial development, leading to their associations with multiple neurological disorders including schizophrenia [69–71]. Decreased phosphatidylinositol phospholipid levels as well lower expression levels of genes relevant to this signalling pathway were reported in the post-mortem prefrontal cortex of schizophrenia patients [72, 73]. Interestingly, phosphatidylinositol signalling can activate focal adhesion kinase - a central signalling component of focal adhesion, linking to the aforementioned cell adhesion processes [74].

Our drug repurposing utilising the disease signature of differential targeting TFs to find compounds that may correct the abnormalities in schizophrenia. This is the first time drug repurposing based on differential targeting networks has been applied in schizophrenia. It should be noted that different repurposing methodologies could produce different results, for example transcriptomics-based versus genetically-driven. Zhang et al. 2019 utilised a different methodology based on genetic-trait associations and CommonMind Consortium data was used for expression quantitative trait loci analysis [75]. In the Zhang et al. study, repurposing for schizophrenia led to one candidate surviving correction for multiple testing, i.e., phenformin – a withdrawn anti-diabetic agent. While the reported impaired glucose homoeostasis of schizophrenia could be relevant to the potential of phenformin in the disorder, antipsychotics have also been widely associated with metabolic abnormalities [76]. It is challenging to determine whether the metabolic traits linked to schizophrenia could be specific to the disorder or the off-target effects of medications. While 5 out of our 10 top drugs were in the list of drugs associated with schizophrenia in Supplementary Fig. 6 from Zhang et al. (khellin, kaempferol, carbachol, vidarabine, benfotiamine) – none of these survived multiple testings (phenformin was the only one that did in Zhang et al. study). While the different strategies for drug repurposing could offer alternatives suiting different data availability, every drug repurposing candidate should be considered carefully with as much validation as possible. In this study, apart from comprehensive literature review, we replicated the primary dataset’s analyses by applying similar methods to two independent post-mortem datasets from CommonMind Consortium and PsychENCODE (details in Supplementary Methods and Results). Our findings revealed a high replication rate, with 94 out of 100 repurposing candidates replicated in at least one dataset. Notably, 49 of these candidates were replicated in all datasets examined. This supports the notion that TF-based network methodologies could improve reproducibility as mentioned above.

Among the top drugs highlighted in Table 1, rimonabant and kaempferol had preclinical evidence supporting beneficial effects for schizophrenia. Rimonabant is an inverse agonist of cannabinoid receptors and has been shown to normalise psychotic-like behaviours in animal models of schizophrenia [77, 78]. Rimonabant, previously approved as anti-obesity drug, was withdrawn from European market in 2008 due to negative psychiatric side effects (depression and anxiety) [79]. Therefore, comorbid depression was part of exclusion criteria in a 16-week randomised controlled trial in 2011 on neurocognitive impairments in schizophrenia. The trial found rimonabant improved specific learning deficit based on response to positive feedback with no significant difference in anxiety/depression subscale of Brief Psychiatric Rating Scale (BPRS) score [80]. Kaempferol, a polyphenol, has exhibited neuroprotection in rat models of hippocampal damage and memory deficits via the activation of SIRT1 – a neuroprotective gene in schizophrenia [81–83]. Alendronic acid, an osteoporosis medication, has been also highlighted as a repurposing candidate for schizophrenia in another study using a drug-protein interactome [84]. It has been demonstrated that alendronic acid inhibits acetylcholinesterase (AChE) and markedly reduces AChE activity in the frontal cortex of rats [85, 86]. Interestingly, a Cochrane review of clinical randomised trials revealed that the addition of acetylcholinesterase inhibitors to antipsychotics leads to improvements in the overall psychopathology, negative symptomatology, and depressive symptoms in individuals diagnosed with schizophrenia [87]. This suggests alendronic acid could be beneficial for schizophrenia via its effect on AChE.

The top drug repurposing candidates with known mechanisms of action tended to affect the main biological processes enriched by the differential TFs. Khellin, kaempferol and ellagic acid likely affect oxidative phosphorylation. Khellin, a phytochemical extracted from *Ammi visnaga*, could rescue mitochondrial dysfunction in common forms of familial Parkinson’s disease (Table 1 of screening study by Mortiboys et al.) [88]. Kaempferol can also reduce oxidative stress [81, 89]. Ellagic acid, a phenolic acid, was found to alleviate clozapine‑induced oxidative stress and mitochondrial dysfunction in cardiomyocytes [90].

With the relevance of the immune response to schizophrenia, targeting pathogens may ameliorate the disorder. Vidarabine, an antiviral mainly used against herpes simplex virus, has been reported to improve a patient’s schizo-affective disorder possibly induced by the viral infection as per a case study reported by Schlitt et al. [91]. Associations of herpes simplex virus to schizophrenia have been found not only in the immediate viral carriers but also in their offspring [92–94].

Benfotiamine and carbachol may be beneficial via phosphatidylinositol signalling. Benfotiamine, a derivative of thiamine, improved cognitive function and suppressed glycogen synthase kinase-3 activity in an animal model of Alzheimer’s disease [95]. Glycogen synthase kinase-3 is a target of Akt, which is a downstream effector of phosphatidylinositol 3-kinase activation [96]. Carbachol, a cholinergic activator, targets M3 muscarinic receptors which enhances phospholipase Cβ 3 in phosphatidylinositol signalling [97]. Cholinergic activation of M3 and M1 receptors induced by carbachol was also found to facilitate synaptic plasticity in a model of GABA dysfunction in schizophrenia [98]. Alizapride (a dopamine 2 receptor antagonist) and glutamine (the main precursor of glutamate) affect the main neurotransmission targets of current antipsychotic drugs [99–101]. While the dopaminergic and glutamatergic pathways were not among the most significantly enriched pathways by differential targeting of TFs, they still had significant nominal *p*-values (Supplementary Table 2). Circulating glutamate and glutamine levels was suggested to be under dual regulatory pattern in schizophrenia. Madeira et al. reported increased glutamine/glutamate ratio versus healthy individuals at the recent onset of schizophrenia followed by a decrease of the ratio in chronic patients [102]. A meta-analysis of ^1^H magnetic resonance spectroscopy studies found higher glutamine in frontal brain region of schizophrenia patients, yet both glutamine and glutamate levels reduced at a faster rate with age comparing with healthy controls [103]. It was unclear if such glutamatergic changes were due to the progression of the disease or antipsychotic usage, making it hard to justify the potential of glutamine for treatment.

There are some limitations of this study. The methodology has not been subjected to benchmarking, due to the lack of suitable ground-truth drug repurposing datasets for sensitivity and specificity analyses. Gene regulatory networks may be biased towards well-studied TFs and proteins. The results depend on limited treatment response data, which could have been yielded from non-neuronal cell types. In addition, the transcriptomics data was not derived from drug-naïve patients, potentially diminishing the importance of the main targets of current medications (e.g., dopamine antagonists) in the drug repurposing results. Nevertheless, the identified repurposing candidates may work on poorly addressed pathological features of schizophrenia, as highlighted by the enriched pathways potentially targeted by them. Only post-mortem samples from DLPFC were considered, hence the results may not be generalisable to other brain regions. The DLPFC focus is due to the various evidence showing abnormalities in schizophrenia from genetics to functional imaging [104–107]. It would be important to examine other brain regions in the future. Furthermore, a hurdle in post-mortem brain analyses lies in the fact that even with the inclusion of explicit, observed covariates, there may still be an incomplete accounting for the effects of RNA degradation or other latent variables [108]. In view of this, the results of this study should be interpreted carefully, as more research is necessary before clinical implementation.

In conclusion, our study deployed comprehensive network-based approaches taking advantage of high-throughput data and prior knowledge to elucidate gene expression regulation driven by TFs in schizophrenia. Energy metabolism, immune response, cell adhesion, and thyroid hormone signalling are among the significant pathways that have been unveiled to be most regulated by the TFs in the disorder. Using the TF signatures from regulatory perturbations in schizophrenia, we ultimately searched for potential drugs that can be repurposed to treat schizophrenia. The best repurposing candidates with known mechanisms were then described in the context of TF-targeted pathways. Those candidates, especially ones with supported preclinical evidence such as kaempferol, should be studied further on their potential mechanisms of action and efficacy in ameliorating schizophrenia.

### Supplementary information

Supplementary Information

Supplementary Table 1-4
