# Supplementary material for: Network-based drug repurposing for schizophrenia
Source: Neuropsychopharmacology. 2024 Feb 6;49(6):983–92. doi: 10.1038/s41386-024-01805-6 (PMC11039639; doi:10.1038/s41386-024-01805-6)
Supplement: Supplementary file 1 — Supplementary Information [file 41386_2024_1805_MOESM1_ESM.pdf]

Supplementary Information

Network-based drug repurposing for schizophrenia

Trang TT Truong[1], Zoe SJ Liu[1], Bruna Panizzutti[1], Jee Hyun Kim[1,2], Olivia M Dean[1,2], Michael Berk[1,2,3], Ken Walder[1]*

1. Deakin University, IMPACT, The Institute for Mental and Physical Health and Clinical Translation, School of Medicine, Geelong, Australia

2. Florey Institute of Neuroscience and Mental Health, Parkville, Australia

3. Orygen, The National Centre of Excellence in Youth Mental Health, Centre for Youth Mental Health, The Florey Institute for Neuroscience and Mental Health and the Department of Psychiatry, University of Melbourne, Parkville 3010, Australia

* Corresponding author

This file includes:

Supplementary Methods

Supplementary Results

References

Supplementary Figures 1-4

Methods

Replication with CommonMind Consortium - HBCC Brain Bank cohort

- RNA sequencing data:

Dorsolateral prefrontal cortex (DLPFC) RNA sequencing data were accessed from the CommonMind Consortium [1]. After quality control, a total of 237 post-mortem samples belonging to the HBCC Brain Bank were collected from 156 unaffected control subjects and 81 people with schizophrenia. Genes being expressed at more than 0.5 count per million (CPM) in at least 30% of samples were kept for downstream analyses.

To generate input for the co-expression network, the R package variancePartition (version 1.26.0) was used to create expression residuals [2]. Covariates (i.e., diagnosis, sex, RNA integrity number, cell type composition, age of death, intronic rate, intragenic rate, intergenic rate, and ribosomal RNA rate) were regressed out from the full model (i.e., excluded effects). Then the main variable of diagnosis and the intercept were added back in to pertain residuals for the downstream comparison (schizophrenia versus controls). The expression residuals underwent pre-processing (removal of genes with no counts, taking the average of duplicated genes), before being calculated for co-expression using Pearson correlations for network construction.

- Gene co-expression regulatory networks

The R package PANDA was used to build the bipartite gene regulatory network that linked transcription factors (TFs) to their target genes [3]. PANDA integrates three sources of information to infer the TF-gene regulatory network: TF physical protein-protein interactions (TF - TF links), gene co-expression (gene - gene links) and TF motif binding sites (TF - gene links) [3].

TF protein-protein interactions (PPI) were obtained from the STRING database [4]. A threshold of 0.7 (high confidence) was applied to the combined score to convert the score to binary (0 implies no interaction and 1 implies high likelihood of interaction). Binding motifs were acquired from previous studies [5,6], where TF binding domain sequences (i.e., motifs) were scanned for their presence in the promoter regions of genes where transcription initiates.

Expression residuals, TF PPI and binding motifs were inputted in PANDA with the following non-default parameters to make sure only mutual connections shared by PPI, co-expression and TF motifs were considered in the networks: mode = “legacy”, remove.missing.motif = True, remove.missing.ppi = True, remove.missing.genes = True. Two separate regulatory networks were built for schizophrenia cases and unaffected control subjects. Edge weight of each network implied the strength of connection of TFs and genes, reflected via Pearson's correlation coefficient between the TF and the target gene.

- Differential schizophrenia network and finding drug repurposing candidates

To find the differences in regulation in schizophrenia patients as compared to unaffected control subjects, the two corresponding regulatory networks were first aligned and filtered to keep intersections of genes and TFs only. Then the differential network was estimated by subtracting the edge weights of the unaffected control network from those of schizophrenia network. The 100 top positively differential TFs and 100 top negatively differential TFs based on the differential targeting score were submitted to the CLUEreg tool of the GRAND database [32] which utilises differential network signatures to find drugs that potentially target the disease’s gene signature.

Replication with PsychENCODE BrainGVEX cohort

- RNA sequencing data:

Dorsolateral prefrontal cortex (DLPFC) RNA sequencing data were accessed from the PsychENCODE [7]. After quality control, a total of 364 post-mortem samples belonging to the

BrainGVEX cohort were collected from 259 unaffected control subjects and 95 people with schizophrenia. Genes being expressed at more than 0.1 transcripts per million (TPM) in at least 25% of samples were kept for downstream analyses.

To generate input for the co-expression network, the R package variancePartition (version 1.26.0) was used to create expression residuals [2]. Covariates (i.e., diagnosis, sex, RNA integrity number) were regressed out from the full model (i.e., excluded effects). Then the main variable of diagnosis and the intercept were added back in to pertain residuals for the downstream comparison (schizophrenia versus controls). The expression residuals underwent pre-processing (removal of genes with no counts, taking the average of duplicated genes), before being calculated for co-expression using Pearson correlations for network construction.

- Gene co-expression regulatory networks

The R package PANDA was used to build the bipartite gene regulatory network that linked transcription factors (TFs) to their target genes [3]. PANDA integrates three sources of information to infer the TF-gene regulatory network: TF physical protein-protein interactions (TF - TF links), gene co-expression (gene - gene links) and TF motif binding sites (TF - gene links) [3].

TF protein-protein interactions (PPI) were obtained from the STRING database [4]. A threshold of 0.7 (high confidence) was applied to the combined score to convert the score to binary (0 implies no interaction and 1 implies high likelihood of interaction). Binding motifs were acquired from previous studies [5,6], where TF binding domain sequences (i.e., motifs) were scanned for their presence in the promoter regions of genes where transcription initiates.

Expression residuals, TF PPI and binding motifs were inputted in PANDA with the following non-default parameters to make sure only mutual connections shared by PPI, co-expression and TF motifs were considered in the networks: mode = “legacy”, remove.missing.motif = True, remove.missing.ppi = True, remove.missing.genes = True. Two separate regulatory networks were built for schizophrenia cases and unaffected control subjects. Edge weight of each network implied the strength of connection of TFs and genes, reflected via Pearson's correlation coefficient between the TF and the target gene.

- Differential schizophrenia network and finding drug repurposing candidates

To find the differences in regulation in schizophrenia patients as compared to unaffected control subjects, the two corresponding regulatory networks were first aligned and filtered to keep intersections of genes and TFs only. Then the differential network was estimated by subtracting the edge weights of the unaffected control network from those of schizophrenia network. The 100 top positively differential TFs and 100 top negatively differential TFs based on the differential targeting score were submitted to the CLUEreg tool of the GRAND database [32] which utilises differential network signatures to find drugs that potentially target the disease’s gene signature.

Results

<u>Overlapping significant repurposing candidates (q-value < 0.05) from the top 100 repurposing candidates of each dataset between three datasets:</u> CommonMind Consortium - MSSM – Pitt – Penn Brain Bank (CMC_MPP_current), CommonMind Consortium - HBCC Brain Bank (CMC_HBCC), PsychENCODE – BrainGVEX

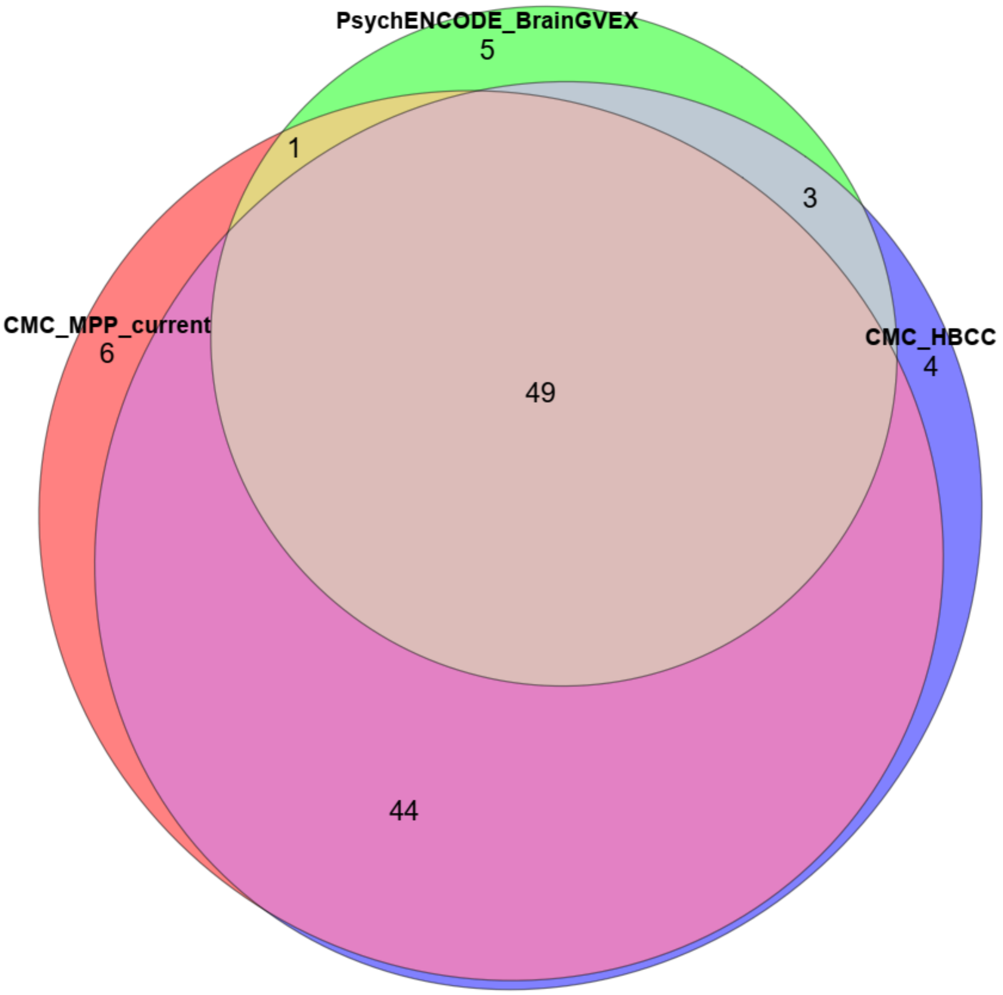

Repurposing candidates from analyses on CommonMind Consortium - HBCC Brain Bank cohort

Drug	Cosine	Q-value
**MD-II-051**	-0.4894	<0.01
**SA-1473648**	-0.4625	<0.01
**SA-3676**	-0.4568	<0.01
**SA-247615**	-0.4511	<0.01
**6-nitrodopamine**	-0.451	<0.01
**SA-1938820**	-0.4481	<0.01
**SA-246522**	-0.435	<0.01
**Dimenhydrinate**	-0.4181	<0.01
**SA-1938862**	-0.4072	<0.01
**SA-85268**	-0.4044	<0.01
**Aminobenztropine**	-0.4032	<0.01
**Rimonabant**	-0.4027	0.0096
**Alendronic-acid**	-0.3967	<0.01
**SA-1939891**	-0.3925	<0.01
**BG-1003**	-0.3882	<0.01
**SA-90153**	-0.3871	<0.01
**SA-96835**	-0.387	<0.01
**SA-1938421**	-0.3859	<0.01
**KDM-103**	-0.3843	<0.01
**Glutamine**	-0.3828	<0.01
**SA-423131**	-0.382	<0.01

**Alizapride**	-0.381	<0.01
**Iniparib**	-0.3784	<0.01
**SA-1938757**	-0.3668	0.045
**SA-96805**	-0.3663	<0.01
**WAY-405**	-0.352	<0.01
**SA-1459224**	-0.351	<0.01
**SA-1920756**	-0.3485	<0.01
**Ciproxifan**	-0.3473	<0.01
**SA-1938867**	-0.3415	<0.01
**Vidarabine**	-0.3402	<0.01
**LBH-589**	-0.3382	<0.01
**PD-168077**	-0.3378	<0.01
**Khellin**	-0.3343	<0.01
**Kaempferol**	-0.3331	<0.01
**SA-427069**	-0.3291	0.0483
**SA-1920722**	-0.3262	<0.01
**KW-06**	-0.3258	<0.01
**SA-90024**	-0.3239	<0.01
**SA-245650**	-0.3235	0.0483
**Allantoxanamide**	-0.3229	<0.01
**SA-1459167**	-0.3215	<0.01
**RAN-02**	-0.3215	<0.01
**SA-1944378**	-0.3209	0.0272
**BG-1001**	-0.3191	<0.01
**BG-1002**	-0.3132	<0.01
**Fluoro-SAHA**	-0.3116	<0.01
**SR-147778**	-0.3105	<0.01
**SA-424755**	-0.3092	0.0483
**1-methylisoquinoline**	-0.3056	<0.01
**Hydroxytyrosol**	-0.3037	0.0483
**SIB-1508Y**	-0.2999	0.0483
**SA-1457169**	-0.2972	0.031
**SA-85377**	-0.2951	0.0214
**KDM-096**	-0.2945	0.0178
**SA-425929**	-0.2889	<0.01
**SR-12813**	-0.2876	0.0178
**Vanillyl-glycol**	-0.2838	<0.01
**WAY-100635**	-0.2797	<0.01
**Epidepride**	-0.2747	<0.01
**Trichloroethylene**	-0.2723	<0.01
**OM-137**	-0.2717	<0.01
**Ellagic-acid**	-0.2697	0.0034
**Bietaserpine**	-0.2695	0.0247
**Tetraethylenepentamine**	-0.2691	<0.01
**SA-427137**	-0.2643	0.0194
**SA-1456305**	-0.2639	0.0483
**SA-1456220**	-0.2638	0.0247
**SA-1459187**	-0.2621	<0.01
**SA-1937516**	-0.25	<0.01
**SA-1459002**	-0.2498	<0.01

**Benfotiamine**	-0.2493	0.0304
**BG-1004**	-0.2489	0.0214
**Desmethyl-DASB**	-0.2473	<0.01
**Carbachol**	-0.2464	<0.01
**Etanidazole**	-0.2427	<0.01
**SA-1463838**	-0.2396	0.0483
**SA-247508**	-0.2366	0.0483
**Chrysamine-g**	-0.2251	<0.01
**SA-244466**	-0.2151	<0.01
**Apramycin**	-0.2136	<0.01
**SA-1459430**	-0.2101	0.0483
**SA-102787**	-0.1978	0.0483
**APEC**	-0.1943	0.0483
**SA-1459256**	-0.1933	0.0483
**Ioversol**	-0.1791	<0.01
**SA-90471**	-0.1784	<0.01
**Nadifloxacin**	-0.1736	0.0483
**SA-1456576**	-0.1684	0.0483
**N-bromoacetyltryptamine**	-0.1659	<0.01
**Nifenazone**	-0.1605	0.0483
**SA-427730**	-0.1553	0.0483
**SA-419021**	-0.1516	0.0483
**Levisoprenaline**	-0.1446	0.0483
**SA-1939064**	-0.1347	0.0096
**Amifostine**	-0.1333	0.0034
**SA-89705**	-0.1249	<0.01
**SA-427228**	-0.1238	<0.01
**SA-1456734**	-0.1094	<0.01
**SA-1456227**	-0.0931	0.0483

Repurposing candidates from analyses on PsychENCODE BrainGVEX cohort

**Drug**	**Cosine**	**Q-value**
**SA-1938421**	-0.4908	< 0.01
**Dimenhydrinate**	-0.4412	0.0096
**Alizapride**	-0.4232	< 0.01
**Rimonabant**	-0.2943	< 0.01
**SA-1473648**	-0.2861	< 0.01
**Iniparib**	-0.2825	0.1248
**SA-85268**	-0.2821	< 0.01
**Flumethasone**	-0.2778	< 0.01
**Glutamine**	-0.2748	< 0.01
**Etiocholanolone**	-0.2643	0.0532
**Flurandrenolide**	-0.2592	< 0.01
**SA-1938820**	-0.2467	< 0.01
**KDM-103**	-0.2451	< 0.01
**Famprofazone**	-0.2377	0.1248
**MD-II-051**	-0.2361	0.1248
**Ciproxifan**	-0.2327	< 0.01
**Dichlorodiamine-platinum**	-0.2236	0.1248

**SA-1938757**	-0.2212	< 0.01
**Fluoro-SAHA**	-0.2153	0.1248
**SA-247615**	-0.2141	0.0462
**SA-96805**	-0.2092	< 0.01
**KDM-096**	-0.2089	< 0.01
**SR-147778**	-0.208	0.1248
**Dihydro-7-desacetyldeoxygedunin**	-0.2047	0.1214
**SA-96835**	-0.2029	< 0.01
**Alendronic-acid**	-0.2017	< 0.01
**SA-427069**	-0.2016	0.1512
**SA-246522**	-0.2002	< 0.01
**SA-427137**	-0.1992	< 0.01
**SA-1944378**	-0.1935	< 0.01
**LBH-589**	-0.1912	< 0.01
**Apramycin**	-0.1908	< 0.01
**Hydroxytyrosol**	-0.1885	< 0.01
**SIB-1508Y**	-0.1871	0.0049
**1-methylisoquinoline**	-0.1855	< 0.01
**6-nitrodopamine**	-0.1844	< 0.01
**Bietaserpine**	-0.1832	0.088
**Vanillyl-glycol**	-0.1825	< 0.01
**Pramocaine**	-0.181	< 0.01
**Epidepride**	-0.1809	< 0.01
**SA-1939891**	-0.1802	< 0.01
**Carbachol**	-0.1781	< 0.01
**SA-1938862**	-0.1775	< 0.01
**Tetraethylenepentamine**	-0.1758	0.1512
**SA-1459002**	-0.1734	< 0.01
**SA-89735**	-0.173	0.0137
**Benfotiamine**	-0.1728	0.1248
**RAN-02**	-0.1705	< 0.01
**PD-168077**	-0.1654	0.1542
**SA-1480001**	-0.1643	0.1248
**SA-1459224**	-0.1614	0.1248
**SA-85377**	-0.1601	0.1248
**SA-423131**	-0.16	< 0.01
**Nadifloxacin**	-0.1571	< 0.01
**BG-1003**	-0.1568	0.0532
**Etanidazole**	-0.1556	< 0.01
**Vatalanib**	-0.1543	< 0.01
**WAY-100635**	-0.1541	0.1697
**SA-1938867**	-0.1526	0.0096
**SA-1459187**	-0.1515	0.1512
**SA-3676**	-0.1512	0.1248
**Trichloroethylene**	-0.151	0.1512
**Hexachlorophene**	-0.1477	0.1697
**SA-1459167**	-0.1454	< 0.01
**Kaempferol**	-0.1451	0.1697
**Aminobenztropine**	-0.1432	< 0.01
**KW-06**	-0.1403	< 0.01

**SA-419021**	-0.1393	0.1697
**SA-1458026**	-0.1391	0.1248
**SA-425929**	-0.1383	0.1218
**SA-1937516**	-0.1357	0.1542
**SA-427730**	-0.1355	< 0.01
**Levopropoxyphene**	-0.1354	< 0.01
**Khellin**	-0.1344	0.1217
**SA-90153**	-0.134	0.0178
**Lactotensin**	-0.1299	0.0965
**SA-245650**	-0.1255	< 0.01
**WAY-405**	-0.1234	0.1512
**Nifenazone**	-0.1224	0.0431
**SA-424755**	-0.122	0.1697
**Ioversol**	-0.12	0.1512
**SA-1939064**	-0.1179	0.1697
**Desmethyl-DASB**	-0.117	< 0.01
**SA-90471**	-0.1132	0.1697
**BG-1002**	-0.1127	< 0.01
**SA-247508**	-0.1106	< 0.01
**SA-1920756**	-0.1075	0.1697
**BG-1001**	-0.0966	0.1512
**SA-1457169**	-0.0934	< 0.01
**SA-427228**	-0.0933	0.1697
**N-bromoacetyltryptamine**	-0.0932	0.1697
**SA-89705**	-0.0837	< 0.01
**SA-90024**	-0.083	< 0.01
**Ellagic-acid**	-0.078	< 0.01
**Allantoxanamide**	-0.0776	0.1697
**Levisoprenaline**	-0.0772	< 0.01
**SA-1920722**	-0.0762	0.1697
**APEC**	-0.0681	0.1697
**SA-1456220**	-0.0551	< 0.01
**SA-1456305**	-0.053	0.1512

References

1. Hoffman, G.E.; Bendl, J.; Voloudakis, G.; Montgomery, K.S.; Sloofman, L.; Wang, Y.-C.; Shah, H.R.; Hauberg, M.E.; Johnson, J.S.; Girdhar, K.; et al. CommonMind Consortium provides transcriptomic and epigenomic data for Schizophrenia and Bipolar Disorder. *Scientific Data* **2019**, *6*, 180, doi:10.1038/s41597-019-0183-6.
2. Hoffman, G.E.; Schadt, E.E. variancePartition: interpreting drivers of variation in complex gene expression studies. *BMC Bioinformatics* **2016**, *17*, 483, doi:10.1186/s12859-016-1323-z.
3. Glass, K.; Huttenhower, C.; Quackenbush, J.; Yuan, G.-C. Passing Messages between Biological Networks to Refine Predicted Interactions. *PLOS ONE* **2013**, *8*, e64832, doi:10.1371/journal.pone.0064832.
4. Szklarczyk, D.; Gable, A.L.; Nastou, K.C.; Lyon, D.; Kirsch, R.; Pyysalo, S.; Doncheva, N.T.; Legeay, M.; Fang, T.; Bork, P.; et al. The STRING database in 2021: customizable protein–protein networks, and functional characterization of user-uploaded gene/measurement sets. *Nucleic Acids Research* **2021**, *49*, D605-D612, doi:10.1093/nar/gkaa1074.
5. Lopes-Ramos, C.M.; Chen, C.-Y.; Kuijjer, M.L.; Paulson, J.N.; Sonawane, A.R.; Fagny, M.; Platig, J.; Glass, K.; Quackenbush, J.; DeMeo, D.L. Sex Differences in Gene Expression and Regulatory Networks across 29 Human Tissues. *Cell Reports* **2020**, *31*, 107795, doi:https://doi.org/10.1016/j.celrep.2020.107795.

6. Weirauch, M.T.; Yang, A.; Albu, M.; Cote, A.G.; Montenegro-Montero, A.; Drewe, P.; Najafabadi, H.S.; Lambert, S.A.; Mann, I.; Cook, K.; et al. Determination and inference of eukaryotic transcription factor sequence specificity. *Cell* **2014**, *158*, 1431-1443, doi:10.1016/j.cell.2014.08.009.
7. Akbarian, S.; Liu, C.; Knowles, J.A.; Vaccarino, F.M.; Farnham, P.J.; Crawford, G.E.; Jaffe, A.E.; Pinto, D.; Dracheva, S.; Geschwind, D.H.; et al. The PsychENCODE project. *Nature Neuroscience* **2015**, *18*, 1707-1712, doi:10.1038/nn.4156.

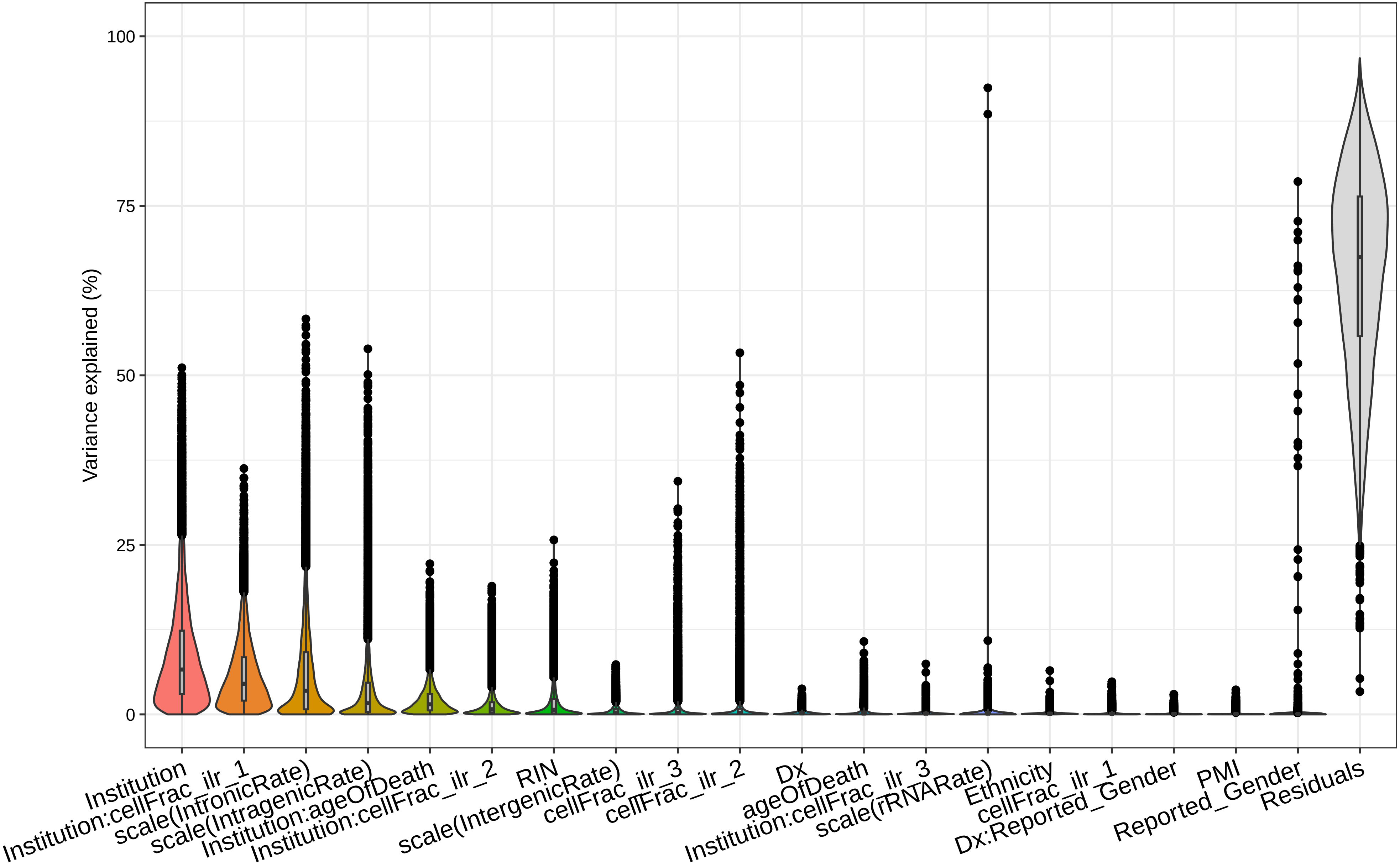

Supplementary Figure 1. Percentage of variance explained by each variable to the total variance of gene expression

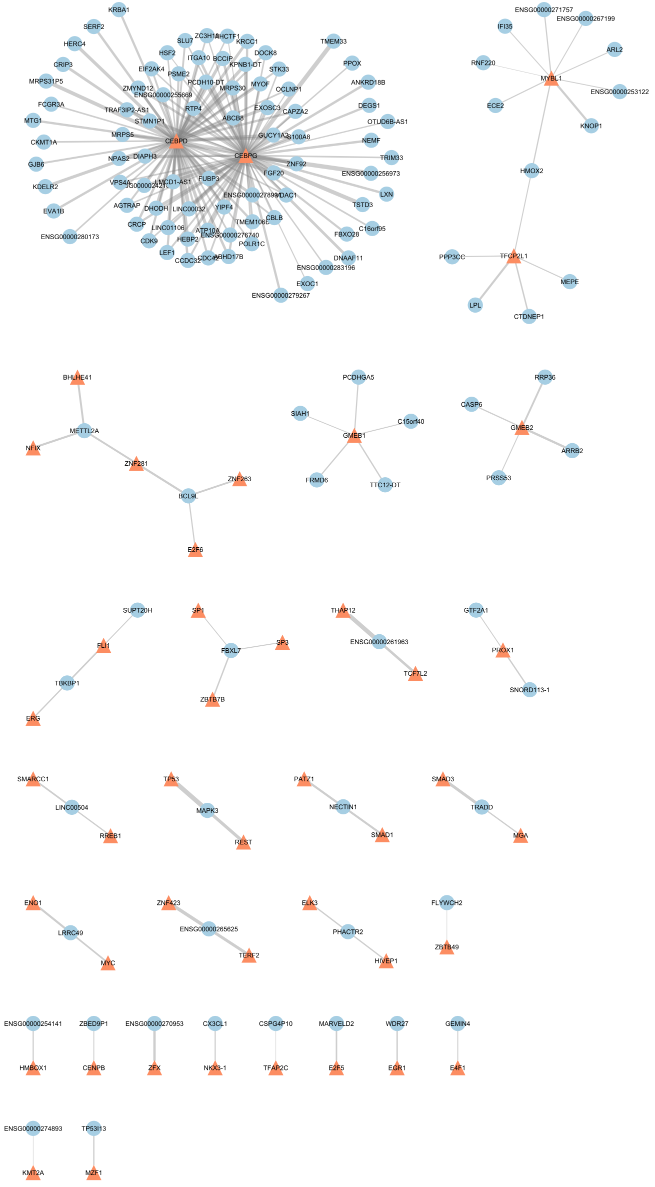

Supplementary Figure 2. Gene regulatory network with top 200 edges of schizophrenia cases

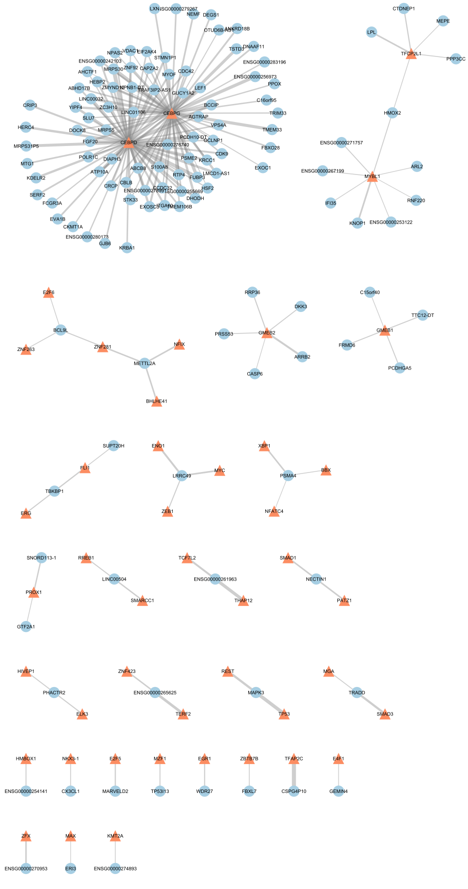

Supplementary Figure 3. Gene regulatory network with top 200 edges of healthy controls

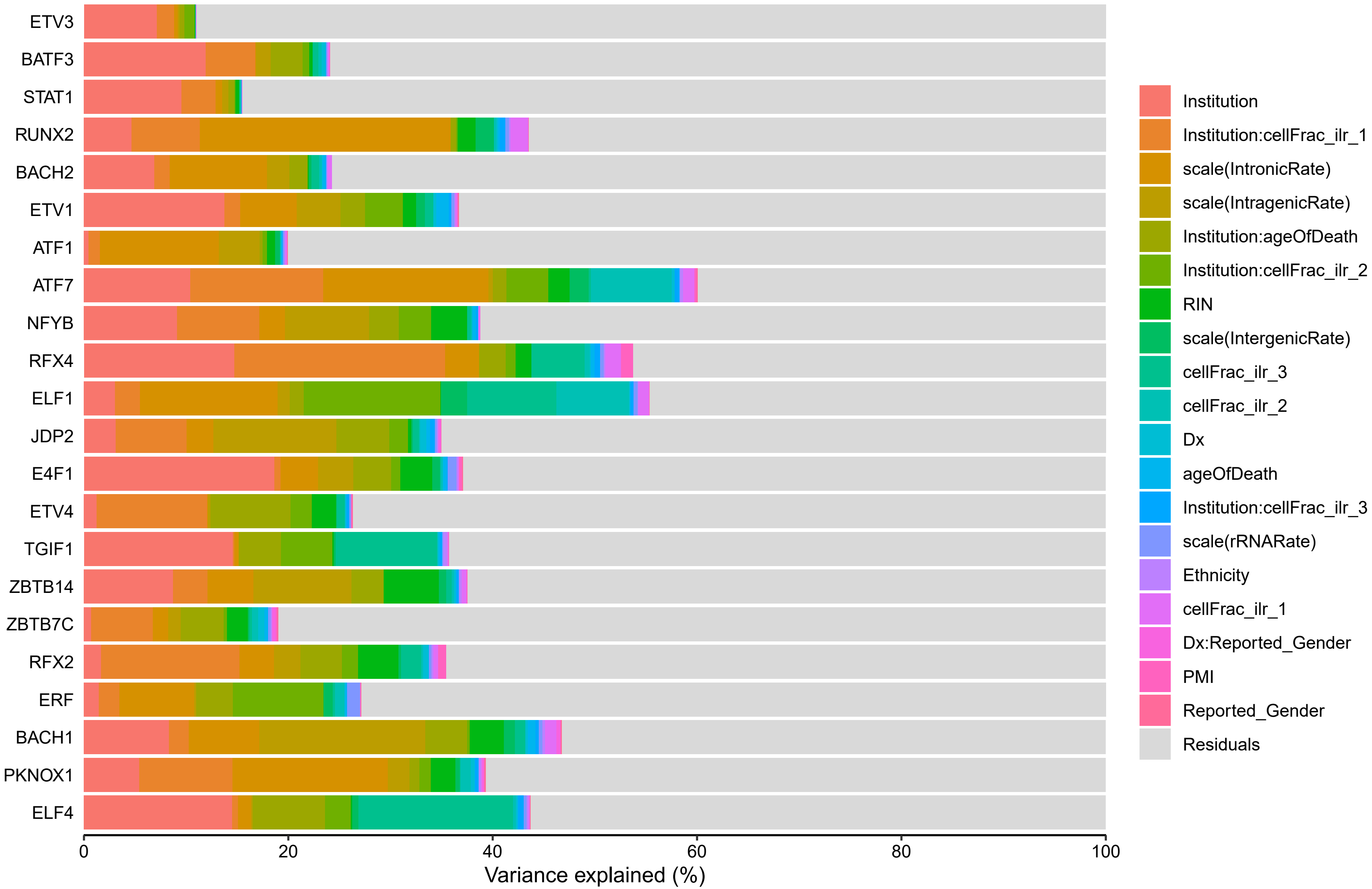

Supplementary Figure 4. Variance partitioned on the variables accounted in linear mixed model for transcription factors with significant differential targeting between schizophrenia and healthy control subjects
